# Differentially Expressed Genes Associated with Improved Drought Tolerance in Creeping Bentgrass Overexpressing a Gene for Cytokinin Biosynthesis

**DOI:** 10.1371/journal.pone.0166676

**Published:** 2016-11-17

**Authors:** Emily Merewitz, Yi Xu, Bingru Huang

**Affiliations:** 1 Department of Plant, Soil, and Microbial Sciences, Michigan State University, East Lansing, 48824, United States of America; 2 Department of Plant Biology and Pathology, Rutgers University, New Brunswick, NJ, 08901, United States of America; New Mexico State University, UNITED STATES

## Abstract

Transformation with an isopentenyl transferase (*ipt*) gene controlling cytokinin (CK) synthesis has been shown to enhance plant drought tolerance. The objective of this study was to identify differentially-expressed genes (DEGs) in creeping bentgrass (*Agrostis stolonifera*) overexpressing *ipt* compared to non-transgenic plants. The *ipt* transgene was controlled by a senescence-activated promoter (*SAG12*). Both a null transformed line (NT) and *SAG12-ipt* plants were exposed to drought stress in an environmentally-controlled growth chamber until the soil water content declined to approximately 5% and leaf relative water content declined to 47%, which were both significantly below the well-watered controls. RNA was extracted from leaf samples of both well-watered and drought-stressed plants. Eight sets of subtractive hybridizations were performed for detection of up-regulated and down-regulated genes due to the presence of the transgene and due to drought stress in both NT and transgenic plants. Sequencing analysis revealed the identity of 252 DEGs due to either the transgene and drought stress. Sequencing analysis of 170 DEGs identified genes encoding for proteins that were related to energy production, metabolism, stress defense, signaling, protein synthesis and transport, and membrane transport could play major roles in the improved drought tolerance by overexpressing *ipt* in creeping bentgrass.

## Introduction

Drought stress affects various physiological and metabolic processes, including the induction of leaf senescence which is known to be regulated by plant hormones, such as CK [1]. Increasing CK synthesis through overexpressing a gene encoding isopentyl transferase controlling CK synthesis (*ipt*) has been shown to increase drought tolerance in creeping bentgrass [2–4] and other species [5–7]. *SAG12-ipt* creeping bentgrass exhibits a greater ability to maintain CK content and improvement in major physiological characteristics governing drought tolerance, as manifested by reduced electrolyte leakage, maintenance of higher relative water content, improvement in photosynthetic rate, and reduction in oxidative damage, which provided strong evidence of improved drought tolerance by increasing endogenous CK [2–4]. Rivero *et al*. [8] examined transcript changes in tobacco overexpressing *ipt* and found an increase in transcripts related to chlorophyll biosynthesis, photosynthetic reactions and other pathways. Their results focused on elevated CK levels in transgenic tobacco plants promoting transcription of photosynthesis-associated gene transcripts, ABA and brassinosteroid associated responses. Little discussion of antioxidant enzymes or other stress associated transcripts is available. In creeping bentgrass, enhanced cytokinin content via *SAG-ipt* expression promoted the expression of specific stress responsive proteins such as antioxidants and chaperones [3]. Thus, identifying gene expression changes associated with drought stress and enhanced cytokinin biosynthesis for stress responses, particularly in a monocot grass species is needed. Additionally, the physiology of turfgrass species is significantly different than other species due to the unique nature of their culture, which includes mowing. Mowing has been found to have a significant effect on hormone accumulations in creeping bentgrass [9]. Thus, how cytokinins affect gene expression in a mowed plant deserves investigation.

Proteomic evaluation of *SAG12-ipt* creeping bentgrass revealed major changes in the proteome during drought stress compared to NT plants. Protein abundance and activity assays revealed that enhanced CK may elicit improved physiological characteristics associated with a reduction in leaf senescence and drought damage via maintenance of greater antioxidant enzyme activities (e.g. superoxide dismutase, peroxidases, 2-Cys peroxiredoxin, and catalase). In addition to stress protective proteins, proteins associated with major metabolic pathways such as energy production, carbon and nitrogen metabolism, protein synthesis and destination were generally maintained to a greater extent *SAG12-ipt* plants compared to the null transformed line (NT) [4]. Metabolomic analysis revealed similar metabolic changes to those found in proteomic analysis, as major metabolites such as carbohydrate, amino acids, and organic acids involved carbohydrate metabolism and energy production pathways accumulated to a greater extent in *SAG12-ipt* plants compared to NT [10]. However, the underlying transcript changes associated with the aforementioned metabolic processes found through metabolic and proteomic profiling due to *ipt* overexpression that could contribute to the improved drought tolerance have yet to be documented. Identification of gene changes may help in elucidating molecular factors accounting for proteomic and metabolic changes contributing to enhanced drought tolerance by CK.

Subtractive suppressive hybridization (SSH) remains to be an effective way to identify specific differentially-expressed genes or transcript accumulation for comparisons between different genotypes or environmental conditions, despite recent advances and availability of next-generation gene sequencing technologies [11–13]. SSH evaluation followed by sequence analysis has identified genes that play a role in conferring drought tolerance in a number of crop species such as cotton (*Gossypium arboretum*) [14], soybean (*Glycine max*) [15], and corn (*Zea mays*) [16]. In grass species, SSH has been performed to identify heat-responsive genes in Fescue sp. (*Festuca* sp.) [17] and drought stress in perennial ryegrass (*Lolium perenne*) [18]. In drought tolerant compared to sensitive accessions of perennial ryegrass, major differences detected during drought stress were associated with plant antioxidant systems [18]. Numerous drought-responsive genes have been identified through SSH and the next-generation sequencing, as mentioned above, which provides insights into molecular mechanisms of drought responses; however, transcripts altered by increasing CK through genetic transformation that contribute to improved drought tolerance are not well understood. Therefore, the objectives of the study were to identify differentially-expressed genes in creeping bentgrass overexpressing *SAG12-ipt* compared to the NT under both well-watered and drought stressed conditions using SSH analysis and to determine major metabolic processes involved in CK-regulation of drought tolerance.

## Materials and Methods

### Plant material

A null transformed line of creeping bentgrass ‘Penncross’ (NT) and a transgenic line containing the *ipt* gene linked to the *SAG12* promoter (*SAG12-ipt* plants) were exposed to different watering treatments in an environmental growth chamber. The *ipt* gene was cloned from agrobacterium (*Agrobacterium tumefaciens*) and *SAG12-ipt* plants were transformed using the *Agrobacterium* method as described previously [2–4, 19–20]. In order for ease of comparison to previous results, the same plant material used for proteomic and metabolomic evaluation [3–4] was used for SSH analysis. Briefly, plants were vegetatively propagated by separating tillers in a greenhouse. The plants were grown in PVC tubes (40 cm in height x 10.16 cm in diameter) all containing an equal volume of 1:1 fine sand:soil mix (fine-loamy, mixed mesic Typic Hapludult type soil). Plants were watered and fertilized to optimum levels for creeping bentgrass growth in a greenhouse until a full canopy covering the 10.16 cm diameter was achieved. Subsequently, the plants were moved to a controlled-environment growth chamber (Conviron, Winnipeg, Canada). All plants were fertilized once per week with Hoagland’s nutrient solution [21] and grown in growth chamber conditions consisting of 20/15°C (day/night) temperatures, 12 h photoperiod, 60% relative humidity, and 500 μmol m^-2^ s^-1^ photosynthetic photon flux density at canopy height. After a 10 d acclimation period, watering treatments were imposed on 3 March 2009.

### Watering treatments

Both NT and *SAG12-ipt* plant types were subjected to either well-watered control or water stress conditions (total 80 plants). A subset of 20 plants of NT and *SAG12-ipt* were watered once daily to maintain soil volumetric water content (SWC) at approximately 25–30% (well-watered control). SWC was determined with the Time Domain Reflectometry (TDR) method [22] using a Trase TDR instrument (Soil Moisture Equipment Corp., Santa Barbara, CA). SWC was measured with one three-pronged waveguide probes (20 cm in length, spaced 2.54 cm apart) installed vertically in each pot, four probes in the control treatment and four probes in the water stress treatment (four replicates in each line). Pot capacity of the soil water was approximately 25%. Water stress was imposed to a subset of 20 plants of each plant type by completely withholding irrigation. The well-watered control and drought treatments each included four replicates for the NT and transgenic line.

### cDNA library construction and SSH analysis

Leaves collected from NT and *SAG12-ipt* plants maintained under well-watered conditions and drought stress with 47% RWC were used to extract total RNA. For total RNA extraction, leaf tissue was ground to a fine powder with mortar and pestle while constantly in the presence of liquid nitrogen. Total RNA was then extracted from the ground leaf tissue with Trizol Reagent, following the manufacturer’s instructions (Invitrogen, Carlsbad, CA, USA). The poly adenylated mRNA fraction was isolated from total RNA using an Oligotex isolation midi kit (Qiagen, Valencia, CA, USA). Total RNA content and mRNA concentrations were evaluated using a spectrophotometric method in a NanoDrop instrument (ThermoScientific Co, USA). DNase-treated total RNA (Turbo DNA-free kit, Ambion Inc., Austin, TX) was used for the SMARTer PCR cDNA synthesis kit in order to generate 4 ug of mRNA from each sample. The mRNA was then utilized in the PCR-Select cDNA subtraction kit (Clontech, Takara BIO, Inc., Mountainview, CA) using the protocols provided by the manufacturer. The PCR-Select cDNA subtraction kit was used to generate both forward and reverse subtraction libraries indicating differential expression between the NT and *SAG12-ipt* plants under both well-watered and drought stressed conditions (47% RWC). A total of 16 cDNA libraries were created in order for performing 16 subtractive hybridizations to generate the subtracted libraries [4 treatments x 2 libraries (forward and reverse) x 2 repetitions]. The treatment expected to contain mRNA of the more drought tolerant or less stressed type (e.g. well-watered treatment or transgenic line) were utilized as testers in the hybridizations and the more sensitive were used as drivers (e.g. drought stressed plants or NT). For instance, in the subtraction comparing both NT and *SAG12-ipt* under drought stressed conditions, the forward subtraction identified mRNA transcripts from the drought-stressed *SAG12-ipt* plants was used as the tester and the mRNA from NT was used as the driver.

### Gene cloning, sequence analysis, and experimental design

Plants were arranged as appropriate for a split-plot design with water treatment as the main plots and plant materials as the sub-plots, with four replicates for each water treatment and plant material. Sequences obtained were evaluated and gene identity matches were selected for those that had a significantly low e value (e value < 10^−5^). A cloning procedure was performed with competent cells based on the manufacturer’s instructions (TOPO-TA cloning kit, Life Technologies, Carlsbad, CA). Colonies were plated in glycerol stock and kept at -80°C until sequencing. Colonies were selected and sent for sequencing utilizing M13 reverse primers (Genewiz, South Plainfield, NJ). Gene sequences were categorized based on their predicted function based on the system used previously in Bevan *et al*. [23] and [2–3]. Sequences were evaluated to remove redundant base pairs and vector sequences manually and through use of VecScreen BLAST database. Clean Blastnr and BLAST of EST databases were performed [24].

### qPCR Analysis

qPCR confirmation of SSH analysis was performed on samples taken from WT and *SAG12-ipt* plants within a separate experiment. Genes were selected for qPCR out of the total sequenced forward and reverse libraries based on relevance to the proteomics, metabolomics, and SSH results. The experimental details and conditions are as follows: Leaf samples were ground in liquid nitrogen using pre-chilled mortar and pestle for four biological replicates. Total RNAs were extracted from all samples of leaf powder using TRIzol reagent and treated with DNase (TURBO DNA-free kit, Life Technologies, Carlsbad, CA). The quality and quantity of RNA were assessed with NanoDrop 1000 (Thermo Scientific, DE). 2 μg of RNA was used for cDNA synthesis using High-Capacity cDNA Reverse Transcription kit (Life Technologies, NY). PCR amplification was carried out using gene-specific primers and SYBR green master mix (Life Technologies, Carlsbad, CA), according to manufacturer’s instruction. Relative transcript abundance was calculated using Δ Δ Ct method with actin as an internal standard. Primers used are listed in Table 1.

**Table 1:** Primer sequences used in qPCR analysis of creeping bentgrass leaf tissues. The primer design tool feature of BLAST was utilized based on sequences obtained from SSH analysis.

Description	primer F	primer R
Actin	GATATGGAAAAGATCTGGCATCAC	TCATACTCGGCCTTGGAGATCCAC
LRR receptor-like kinase 1	AGGGACCATACCTCCCATTC	CAGGTCTTGACACTGGCTCA
RuBisCo	GCTGCCGAATCTTCTACTGG	AGAGCACGTAGGGCTTTGAA
universal stress protein 5327	GCAGGGACATGGGTGGGAGGA	GTACCCCGCTCTGATGCGCC
LRR receptor-like kinase 2	GCCGTTTTGTGCGCTCCTGT	GGAGATCCAAAGCCGCCACCC
Oxygen-evolving enhancer protein 3–1, chloroplast precursor (OEE3)	AGGGCCTCCTACCTGCGCTAC	TGCCGGTGAGGTCCTTGAGGC

## Results

**Fig 1:**
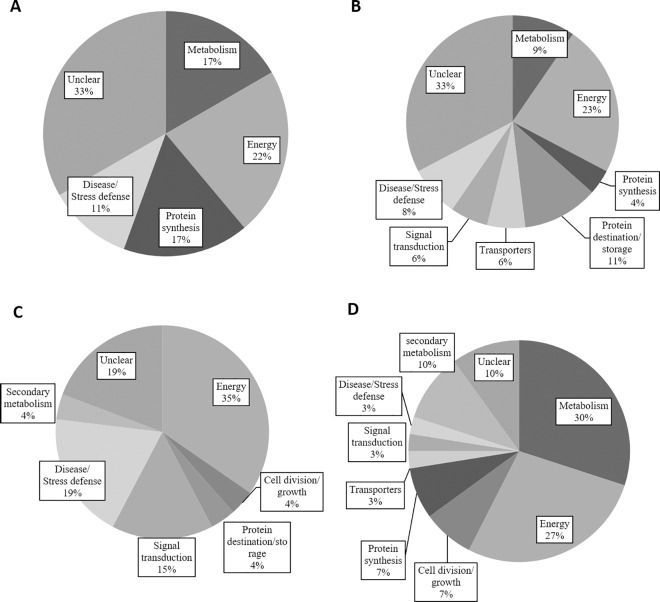
Percent total changes of gene transcripts (including forward and reverse libraries) in functional categories comparing A) NT to *SAG12-ipt* under well-watered conditions B) NT watered to NT drought stress C) *SAG12-ipt* well-watered to *SAG12-ipt* drought stress D) NT drought to *SAG12-ipt* drought stress conditions

Comparing across hybridization libraries or genes that may have marginally differential gene expression can be difficult due to the potential for gene transcript loss during the experimental procedure and general limitations of sensitivity of the SSH approach. Therefore, specific changes will be discussed and interpreted only as they relate to the respective hybridization. The cDNA library construction and SSH subtraction successfully revealed 252 gene transcripts that were alternately regulated due to the *ipt* transgene or watering treatment. Of which, a total of 170 clones were selected for sequencing. After removal of redundant sequences and those that were unreadable and BLAST database searches, approximately 136 sequences were successfully identified in the GenBank database and grouped into functional categories (Fig 1). Differential gene regulation of 18 sequences occurred under well-watered conditions due to the *ipt* transgene, which were considered as *ipt*-responsive genes, including genes involved in the metabolism, energy, protein synthesis, stress defense, and unknown categories (Table 2). A total of 49 genes in the forward and reverse libraries comparing NT plants under the well-watered and drought stress condition were successfully sequenced (Table 3). For the *SAG12-ipt* libraries comparing well-watered to drought stress conditions, 26 genes exhibited differential expression (Table 4). A total of 75 genes were identified as drought-responsive genes in NT plants (49 genes) and *SAG12-ipt* plants (26 genes). The results of the libraries comparing NT and *SAG12-ipt* plants both under drought stress were a total of 38 genes being alternately regulated, including those that were up-regulating and down-regulated (Table 5).

**Table 2:** Genes categorized by protein function based on BLAST searches that were differentially expressed in SSH library A (NT compared to *SAG12-ipt* transgenic plants both growing under well-watered conditions) in the forward (up-regulated by *ipt* expression) and reverse (down-regulated by *ipt* expression) libraries. Numbers in parentheses indicate number of transcript isoforms detected.

Library	Description	Accession #	Best Match Accession #	E-value
**Category 01 Metabolism**				
Forward	β-glucosidase	JZ948409	ACF22735.1	6.00E-40
**Category 02 Energy**				
Forward	enolase (2-phosphoglycerate dehydratase)	JZ948410	AAM69295.1	3.00E-60
	photosystem I P700 chlorophyll a apoprotein A2	JZ948411	ABG66207.1	1.00E-71
	photosystem II precursor, chloroplast	JZ948412	NP_001134061.1	9.00E-68
	RuBisCO large subunit (3)	JZ948413	ADU18941.1	4.00E-98
		JZ948414	ADU18941.1	5.00E-49
		JZ948415	ADU18941.1	3.00E-76
Reverse	Glyceraldehyde 3-phosphate dehydrogenase	JZ948416	ACV86034.1	2.00E-133
**Category 05 Protein synthesis**				
Forward	elongation factor 1-alpha-like protein	JZ948417	ABB16977.1	2.00E-79
	nonribosomal peptide synthetase	JZ948418	CBJ23772.1	1.00E-10
	40S ribosomal protein S21	JZ948419	NP_001105477.1	1.00E-47
**Category 10 Signal transduction**				
Forward	protein phosphatase 1 regulatory subunit 12b	JZ948420	XP_001662854.1	4.00E-18
**Category 11 Disease/Stress defense**				
Forward	catalase 2	JZ948421	A55092	1.00E-08
	catalase	JZ948422	Q59296.1	3.00E-10
**Category 20 Secondary metabolism**				
	oleosin 2	JZ948423	NP_198858.1	1.00E-76
	S-adenosylmethionine synthase 4 (SAMS)	JZ948424	Q4LB21.1	2.00E-60
**Category 12 Unclear**				
Forward	predicted protein	JZ948425	BAJ87310.1	9.00E-104
	predicted protein	JZ948426	BAJ96581.1	4.00E-117
	predicted protein	JZ948427	BAK02049.1	3.00E-36
	SORBIDRAFT_01g032220 (2)	JZ948428	XP_002467681.1	5.00E-58
		JZ948434	XP_002467681.1	3.00E-37
Reverse	predicted protein	JZ948430	BAJ93277.1	4.00E-16
	predicted: similar to CG18041 CG18041-PA	JZ948431	XP_969418.1	4.00E-18

**Table 3:** Genes categorized by protein function based on BLAST searches that were differentially expressed in SSH library B (NT under well-watered conditions compared to NT under drought stressed conditions) in the forward (up-regulated by drought) and reverse (down-regulated by drought) libraries. Numbers in parentheses indicate number of transcript isoforms detected.

Library	Description	Accession #	Best Match Accession	E-value
**Category 01 Metabolism**		** **	** **	** **
Forward	malate dehydrogenase	JZ948432	XP_001659012.1	2.00E-29
	glycogen synthase kinase-3 MsK-3	JZ948433	NP_001148880.1	6.00E-07
Reverse	nitrilase-associated protein, putative	JZ948434	CAJ38376.1	4.00E-12
	thiamine biosythesis protein ThiC	JZ948435	AAG49550.1	1.00E-54
**Category 02 Energy**				
Forward	RuBisCO large subunit (3)	JZ948436	BAD20627.1	2.00E-44
		JZ948437	BAD20627.1	8.00E-60
		JZ948438	BAD20627.1	1.00E-53
	RuBisCO small subunit 1B	JZ948439	NP_198659.1	0.00001
	RuBisCO small chain c	JZ948440	ABR26034.1	0.00013
	Oxygen-evolving enhancer protein 3–1, chloroplast precursor	JZ948441	BAC83128.1	1.00E-37
	cytochrome c oxidase subunit III	JZ948442	YP_003734710.1	8.00E-36
	Photosystem II 10 kDa polypeptide, chloroplast	JZ948443	NP_001134061.1	4.00E-60
	thioredoxin-like 5, chloroplastic	JZ948444	ABR26107.1	3.00E-30
	ATP-citrate synthase, putative	JZ948445	XP_002519229.1	5.00E-49
Reverse	RuBisCO large subunit (3)	JZ948446	ADU18941.1	2.00E-76
		JZ948447	ADU18941.1	2.00E-15
		JZ948448	ADU18941.1	3.00E-08
	RuBisCO	JZ948448	CBF07493.1	1.00E-26
	glyceraldehyde-3-phosphate dehydrogenase 1	JZ948450	ACV86034.1	8.00E-107
	oxygen-evolving enhancer protein 1, chloroplast	JZ948451	ABQ52657.1	9.00E-20
**Category 05: Protein synthesis**				
Forward	elongation factor 1 alpha	JZ948417	AEG78681.1	2.00E-112
**Category 06: Protein destination/storage**				
Forward	E3 ubiquitin-protein ligase UBR5	JZ948540	EFN82877.1	2.00E-57
	thiol disulfide interchange protein txlA	JZ948453	NP_001152226.1	2.00E-06
	GTPase SAR1	JZ948452	ACD03831.1	3.00E-57
Reverse	ubiquitin-60S ribosomal protein L40-like	JZ948454	XP_003465244.1	1.00E-12
	ubiquitin-conjugating enzyme E2-like protein	JZ948455	ADB28900.1	2.00E-14
	ATP-dependent Clp protease ATP-binding subunit clpA	JZ948456	P31542.1	9.00E-34
**Category 07 Transporters**				
Forward	vacuolar H+-ATPase subunit B	JZ948457	BAF38479.1	2.00E-61
	ABC transporter integral membrane protein	JZ948458	ZP_07303354.1	0.00061
	CDGSH iron sulfur domain 1	JZ948459	NP_001004811.1	
**Category 10 Signal transduction**				
Forward	uridine kinase	JZ948460	ZP_00781761.1	0.00042
	serine/threonine kinase receptor precursor-like	JZ948461	BAC57306.1	4.00E-39
Reverse	LRR receptor-like kinase (3)	JZ948462	ACY30448.1	7.00E-70
**Category 11 Disease/Stress defense**				
Forward	jasmonate-induced protein, putative	JZ948463	ABA96835.1	3.00E-08
	universal stress protein 5327	JZ948464	ADB54812.1	4.00E-37
	abscisic acid-responsive HVA22 family protein	JZ948465	XP_002865508.1	3.00E-08
	glyoxalase I	JZ948466	AAW68026.1	1.00E-53
**Category 20 Secondary metabolism**				
Reverse	diphthine synthase, predicted	JZ948467	XP_001604120.1	6.00E-75
**Category 12 Unclear**				
Forward	predicted protein [Hordeum vulgare subsp. vulgare]	JZ948468	BAK02049.1	1.00E-23
	predicted protein [Hordeum vulgare subsp. vulgare]	JZ948469	BAJ85089.1	3.00E-42
	predicted protein [Hordeum vulgare subsp. vulgare]	JZ948470	BAJ96581.1	3.00E-21
	SORBIDRAFT_10g001310	JZ948471	XP_002436374.1	0.00002
	pBMB0558_00760	JZ948472	YP_004169259.1	5.00E-04
	predicted protein [Hordeum vulgare subsp. vulgare]	JZ948473	BAJ95019.1	1.00E-32
	predicted protein [Hordeum vulgare subsp. vulgare]	JZ948474	BAJ99280.1	5.00E-12
	hypothetical protein MELLADRAFT_95019	JZ948475	EGF98952.1	1.00E-23
	predicted protein [Hordeum vulgare subsp. vulgare]	JZ948476	BAJ90401.1	3.00E-16
	hypothetical protein OsJ_26652	JZ948477	EAZ42091.1	6.00E-17
Reverse	predicted protein	JZ948478	XP_001770883.1	0.0004
	hypothetical protein OsJ_19720	JZ948479	EEE64863.1	1.00E-57
	hypothetical protein Tc00.1047053508475.20	JZ948480	XP_804280.1	0.000031
	hypothetical protein LOC100273563	JZ948481	NP_001141453.1	8.00E-12

**Table 4:** Genes categorized by protein function based on BLAST searches that were differentially expressed in SSH library C (*SAG12-ipt* under well-watered conditions compared to *SAG12-ipt* under drought stressed conditions) in the forward (up-regulated by drought) and reverse (down-regulated by drought) libraries. Numbers in parentheses indicate number of transcript isoforms detected.

Library	Description	Accession #	Best Match Accession	E value
**Category 02 Energy**				
Forward	Mg-protoporphyrin IX	JZ948482	CAB58179.1	2.00E-60
	RuBisCo large subunit	JZ948483	ADU18941.1	3.00E-76
	Oxygen-evolving enhancer protein 3–1, chloroplast precursor (OEE3)	JZ948484	BAC83128.1	4.00E-76
	Thioredoxin-like 5, chloroplastic	JZ948485	ABR26107.1	2E-14
Reverse	Fructose-bisphosphate aldolase, class I	JZ948486	AT3G52930	1.00E-109
	Photosystem II 10 kDa polypeptide, chloroplast	JZ948487	NP_001134061.1	2.00E-17
	Cytochrome c oxidase subunit III	JZ948488	ADO60570.1	2.00E-15
**Category 03 Cell growth/division**				
Reverse	tubulin α-3 chain	JZ948489	NP_001167663.1	2.00E-58
**Category 06 Protein destination/storage**				
Reverse	ubiquitin-conjugating enzyme	JZ948490	ADX86831.1	7.00E-51
**Category 10 Signal transduction**				
Forward	HVA22-like protein	JZ948491	JK340582.1	7.00E-31
	F-box/LRR repeat protein	JZ948492	ACY30448.1	5.00E-40
	uridine kinase	JZ948493	ZP_00781761.1	6.00E-30
Reverse	Os01g0629400 (phosphatase-like)	JZ948494	NM_001050175.2	8.00E-60
	ctd-phosphatase-like protein	JZ948495	ABR26130.1	8.00E-60
**Category 11 Stress/disease defense**				
Forward	glyoxalase I (3)	JZ948496	AAW68026.1	4.00E-32
	jasmonate-induced protein, putative	JZ948497	ABA96835.1	5.00E-30
Reverse	DELLA protein RGL1	JZ948541	EG429076.1	0.00E+00
**Category 20 Secondary metabolism**				
Forward	spermidine synthase	JZ948498	AEL33692.1	1.00E-109
**Category 12 Unclear**				
Forward	hypothetical protein NCLIV_068840	JZ948499	CCA30004.1	5.00E-31
	SORBIDRAFT_06g021780	JZ948500	XP_002448130.1	0.011
	OSJNBa0014K14.7	JZ948501	CAE02935.3	2.00E-91
Reverse	hypothetical protein	JZ948502	CAM36311.1	2.00E-08
	predicted protein	JZ948503	BAJ90401.1	5.00E-16

**Table 5:** Genes categorized by protein function based on BLAST searches that were differentially expressed in SSH library D, which compared NT to *SAG12-ipt* under drought stressed conditions equal in cellular water deficit (47% RWC) in the forward (up-regulated by transgene during drought stress) and reverse (down-regulated by transgene during drought stress) libraries. Numbers in parentheses indicate number of transcript isoforms detected.

Library	Description	Accession #	Best Match #	E value
**Category 01 Metabolism **				
Forward	GDP-mannose 3,5-epimerase	JZ948450	XM_003577361.1	3.00E-27
	mannose-1-phosphate guanylyltransferase 3	JZ948505	Q6Z9A3.1	3.00E-149
	UDP-arabinopyranose mutase 1	JZ948506	Q6Z9A3.1	1.00E-15
	glycosyl hydrolase family 19 protein	JZ948507	AAQ84319.1	6.00E-44
	UTP—glucose-1-phosphate uridylyltransferase	JZ948508	Q43772.1	1.00E-15
	UDP-glucose dehydrogenase	JZ948509	AAX08057.1	7.00E-25
	xylose isomerase-like	JZ948510	XM_003562448.1	4.00E-23
	xylose isomerase	JZ948511	CAA64544.1	3.00E-25
Reverse	glycine decarboxylase P subunit	JZ948512	AAB82711.1	8.00E-12
	acetyl-CoA carboxylase	JZ948513	NP_001185143.1	4.00E-21
**Category 02 Energy**				
Forward	RuBisCo large subunit (3)	JZ948514	AAQ08331.1	5.00E-61
		JZ948515	AAQ08331.1	1.00E-26
		JZ948538	AAQ08331.1	2.00E-06
	chloroplast-localized Ptr ToxA-binding protein1	JZ948539	AAR24582.1	6.00E-54
Reverse	RuBisCo large subunit (4)	JZ948516	ACO35581.1	4.00E-46
		JZ948517	ACO35581.1	5.00E-61
		JZ948518	ACO35581.1	2.00E-17
		JZ948519	ACO35581.1	3.00E-76
	thioredoxin-like 5, chloroplastic	JZ948484	ABR26107.1	3.00E-30
	ATP-citrate synthase, putative	JZ948445	XP_002519229.1	5.00E-49
**Category 04 Transcription**				
Forward	partial 16S rRNA gene	JZ948520	FN421445.1	2E-15
	histone H3	JZ948521	XP_001752178.1	1.00E-121
	translational initiation factor eIF1	JZ948522	BAF63490.1	2.00E-11
**Category 05 Protein synthesis**				
Forward	Os07g0609766	JZ948523	NP_001175295.1	3.00E-05
**Category 07 Transporters**				
Forward	aquaporin PIP1-2	JZ948524	NP_001078067.1	1.00E-32
	coatomer subunit beta'-2	JZ948525	NP_175645.1	1.00E-49
Reverse	plasma membrane H+-ATPase	JZ948526	CAC50884.1	1.00E-28
**Category 10 Signal transduction**				
Forward	receptor kinase ORK14	JZ948527	AAM09948.1	1.00E-5.8
**Category 11 Disease/Stress defense **				
Forward	Chloroplast Ptr ToxA-binding protein	JZ948529	AK332987.1	1.00E-45
	glyoxalase I	JZ948528	BAB71741.1	9.00E-26
**Category 20 Secondary metabolism**				
Forward	cruciferin cru4 subunit	JZ948530	X57848.1	2.00E-11
	isoflavone reductase	JZ948531	ACH72670.1	4.00E-57
**Category 12 Unclear**				
Forward	Os11g0169100	JZ948532	NP_001065849.1	7.00E-50
	hypothetical protein	JZ948533	ACG32534.1	5.00E-13
	predicted protein	JZ948534	BAK07943.1	1.00E-41
	predicted protein	JZ948536	BAK05471.1	5.00E-06
Reverse	hypothetical protein	JZ948537	CAM36311.1	8.00E-07

**Fig 2:**
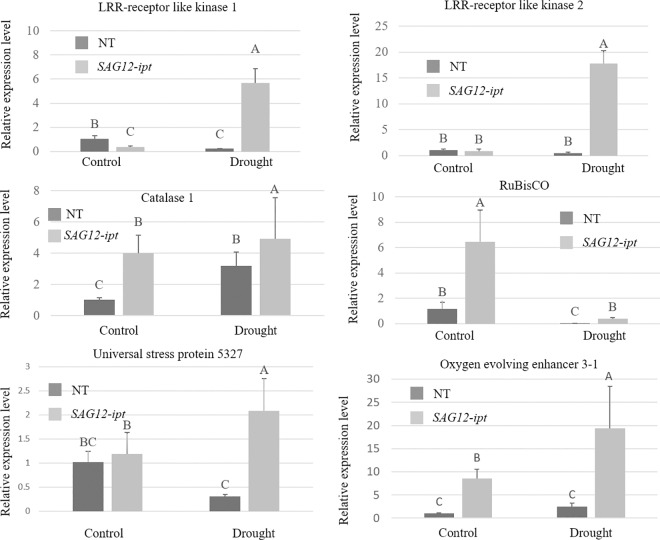
qPCR of creeping bentgrass leaf tissue in null transformed plants (NT) and plants with an *ipt* gene for cytokinin biosynthesis (*SAG12-ipt*) of select genes for confirmation of SSH results. Standard error bars indicate variation among replicates and different letters indicate statistical differences between treatments.

qPCR analysis of transcript levels of selected genes confirmed the validity of SSH screening for differentially-expressed genes between the NT and ipt-transgenic plants in response to drought stress, as results from qPCR were mostly consistent with the gene expression data through SSH analysis (Fig 2). The set of 6 genes with primers are listed in Table 5. Genes such as OEE, LRR receptor like kinase 1, and USP were increased by drought stress in *SAG12-ipt* plants. Elongation factor 1α and a RuBiSCo subunit were decreased by drought stress in both *SAG12-ipt* plants and NT due to drought stress.

## Discussion

The results of SSH analysis have revealed valuable information regarding alterations in gene expression due to the *ipt* gene or drought stress in creeping bentgrass in this study.A large number of DEGs was detected in plants in response to drought stress in this study and previous work has already extensively examined numerous drought-responsive genes [25] particularly in related grass species such as rice (*Oryza sativa*) [26] and wheat [27]. Therefore, the following discussion focuses on those genes that were uniquely up- or down-regulated in the *ipt*-transgenic creeping bentgrass under drought stress (or *ipt*-responsive genes) and that corresponding changes occurred at proteomic and metabolomics levels, as reported in our previous studies [4, 10]. The *ipt*-responsive genes were classified into the functional category of metabolism, energy production, stress defense, transporters, protein metabolism, signaling transduction, and secondary metabolite synthesis. The biological functions of those *ipt*-responsive genes related to drought tolerance are discussed below.

The functional categories having the greatest number of genes differentially expressed in the *ipt*-transgenic plants in the majority of the libraries were in the metabolism and energy production. Proteomic and metabolic analysis also identified changes in proteins and metabolites mainly involved in metabolism and energy production due to the *ipt* overexpression in creeping bentgrass [2–4]. The combined results of gene expression in this study with protein and metabolic data from our previous studies suggest that increased endogenous CK could mainly regulate metabolism and energy production, contributing to improved drought tolerance in the *SAG12-ipt* creeping bentgrass.

SSH results showed that almost twice the number of gene changes occurred due to drought stress in NT plants (52) compared to changes in *SAG12-ipt* plants (26) relative to their respective controls. In addition, NT plants generally had a greater number of gene changes in the metabolism and stress defense categories whereas *SAG12-ipt* exhibited less change in these categories to drought stress. Zhang et al. [17] found that the more heat-tolerant plants of tall fescue (*Poa pratensis* L.) had higher levels of transcripts related to energy production (particularly photosynthesis) protein synthesis, signaling and transcription factors compared to sensitive plants. These data suggested that NT plants that were more sensitive to drought stress exhibited greater transcript changes in metabolism and stress defense categories.

Gene transcripts that were differentially expressed under well-watered conditions due to the *ipt* transgene in the metabolism category had homology to a β-glucosidase gene, SAMS, and a protein phosphatase, which were in greater levels in the *SAG12-ipt* plants compared to NT. The glucosidase proteins are a family of enzymes involved in various cellular functions, including regulating cellular structure, defense, and hormone metabolism [28]. Higher protein abundance of SAMS and protein phosphatase were also detected in *SAG12-ipt* plants compared to NT plants. SAMS activity may be a source of methyl groups for compounds involved in osmotic adjustment under stress conditions [29]. The regulation of SAMS proteins may play a major role in controlling the accumulation of free amino acids or of those destined for proteogenesis, affecting plant growth regulated by CK.

Malate dehydrogenase is a key enzyme in the citric acid cycle, and its transcript level was increased by drought stress in NT plants in this study, but lower accumulation of this protein and malic acid content were detected in NT plants exposed to drought stress in our previous studies [4, 10]. The up-regulation of malate dehydrogenase transcript could function to compensate for the damaged proteins and enzymes induced by drought stress; however, this cannot be directly concluded from the results of this study. Similar results regarding malic acid content have been found in other plant species [30]. The gene for thiamine biosynthesis protein was found to be down-regulated by drought stress only in NT plants in this study. Thiamine is used in the biosynthesis of secondary metabolites such as gamma-aminobutyric acid (GABA). Previous metabolomics analysis found that the content of GABA and other secondary metabolites was lower NT plants compared to that in *SAG12-ipt* plants under drought stress [10], which could be related to the lower transcript level of thiamine biosynthesis in NT plants. Similarly, the differential regulation of other transcripts in the metabolism category such as glycogen synthase and malate dehydrogenase could be associated with the differential sugar accumulation and glycolytic activities detected between NT and *SAG12-ipt* plants [3–4]. Transcripts involved in respiration pathways, such as GAPDH were down-regulated in NT plants. A corresponding decrease in abundance of GAPDH proteins and intermediates in respiratory pathways also occurred in response to drought in NT plants [3]. These results suggested that improved drought tolerance in *SAG12-ipt* plants could be related to CK-regulation of respiratory metabolism involving changes of transcripts controlling synthesis of aforementioned metabolites in respiration pathways; however, more work would be needed to specifically determine *ipt* effects on the metabolites described here.

Differences in carbon requirements and allocation may be a major factor determining the differential drought response of NT and *SAG12-ipt* plants as they relate to carbon metabolism. Drought stress causing an up-regulation of transcripts related to carbon metabolism could be related to carbon availability and partitioning associated with drought damage [31]. Drought stress has also been shown to increase the expression of genes controlling the central processes of carbon metabolism. This is due to a need for carbon mobilization or due to the increased need for replacing damaged enzymes to allow for metabolic processes to remain functional. Increased CK content under drought stress could play a role in regulating carbon availability.

The increase in glycogen synthase kinase (GSK) transcripts in NT plants in response to drought could be associated with an increased need for sugar for stress defense. In rice plants, an increase in GSK was also exhibited due to drought stress [32]. It is not clear why GSK transcript expression was up-regulated while in NT plants a loss of photosynthetic proteins and reduction in photosystem health was observed in our previous studies [4, 10]. GSK may inactivate glycogen synthase or play a role in other metabolic functions such as the inactivation or activation of other transcription factors. Xylose isomerase like protein that exhibited greater transcript level in *SAG12-ipt* transgenic plants compared to NT under drought stress could be directly related to the expression of the *ipt* transgene. O-xylosyltransferases are involved in the regulation of CK hormone biosynthesis via reversible inactivation [33].

Transcripts coding for a Mg-protoporphyrin IX type domain were identified in the forward library of *SAG12-ipt* plants under drought. Based on sequence match results it is not known which enzyme the Mg-protoporphyrin IX domain may be a part of; however, in plants these IX type domains are most commonly a part of the chlorophyll biosynthetic enzyme, Mg-protoporphyrin IX monomethyl ester cyclase [34]. The gene expression and content of this protein is typically reduced by drought stress and maintenance of this enzyme has been associated with improved drought tolerance in transgenic rice [34]. The *SAG12-ipt* plants maintained greater chlorophyll content and photosynthetic activity than NT plants during drought stress [2]. Therefore, the increased expression of these transcripts in the *ipt* plants may be related to chlorophyll maintenance in plants with elevated CK content.

Chloroplast localized ToxA binding protein (Pr ToxA) was detected in the forward library of NT compared to *SAG12-ipt* under drought stressed conditions, meaning a greater number of Pr ToxA were in *SAG12-ipt* plants. Ptr ToxA function involving ToxA are also differentially responsive to cold in frost tolerant and sensitive *Festuca* grass plants and has been found to be required for PSII stability in chloroplasts [35–36]. How Pr ToxA may be related to drought tolerance is not well known. Perhaps, the consistent promotion of PSII health and maintenance of accumulation of photosynthetic compounds in *SAG12-ipt* plants coupled with the increased Pr Tox transcripts under drought stress could be associated with the CK gene regulatory network.

DELLA proteins play a role in various stress responses as they are components of regulatory pathways that are closely associated with plant hormones such as ethylene and gibberellins [37]. A transcript coding for a DELLA protein was reduced by drought stress in *SAG12-ipt* plants. DELLAs are repressors of GA signaling and degradation of DELLA proteins is known to alleviate growth repression [37]. Leaf biomass accumulation under drought stress was doubled in plants containing un-functional DELLA proteins caused by knock-out mutation [38]. Therefore, the reduction of leaf senescence due to CK and the maintenance of metabolic activities in *SAG12-ipt* plants could be related to this differential gene expression of DELLA proteins

Catalase (CAT) is an antioxidant enzyme that detoxifies hydrogen peroxide that can accumulate in plant cells and is an essential enzyme for stress tolerance. CAT expression was greater in *SAG12-ipt* plants compared to NT plants under well-watered condition. Tobacco plants deficient in the CAT enzyme were found to be greatly susceptible to abiotic stress [39]. Enhancement of the expression and activity of CAT promotes stress tolerance [40]. CAT expression in *ipt* transgenic tobacco was enhanced by drought stress to a greater extent than in non-transgenic plants [5]. Several grass species consistently show stable expression of CAT under drought stress [41–42, 3–4]. In addition, leaf senescence is correlated with a decline in CAT activity [43] and CKs have been shown to increase CAT activity [44]. In addition, CAT is also known to stimulate the activity of protoporphyrin cyclase involved in chlorophyll metabolism [45]. The positive roles of CAT for drought tolerance could be due to its antioxidant activity and involvement in chlorophyll metabolism. Therefore, the prevention of natural leaf senescence in the well-watered condition and the maintenance of protein content and activity of CAT under drought stress [3–4] may be a main factor promoting greater drought stress tolerance in *SAG12-ipt* transgenic creeping bentgrass with enhanced CK content. The stability of CAT during drought stress and its great capacity to reduce oxidative stress in grass species may make this a highly effective and valuable molecular target for enhancing or selecting drought tolerant grass germplasm in breeding programs.

Glyoxalase is another stress protective enzyme that accumulates in response to drought conditions in order to break down a toxic by-product of glycolysis, methyl-glyoxyl. In the drought tolerant resurrection grass (*Sporobolus stapfianus*), glyoxylase transcript expression was up-regulated in response to drought [46]. The accumulation of glycolytic proteins [3–4] and transcripts involved in respiration detected in *SAG12-ipt* plants such as GAPDH compared to NT may be related to an increased flux through glycolysis and increased need for the glyoxalase enzyme. In addition, metabolite analysis revealed the accumulation of intermediates in the glycolysis pathway to a greater extent in *SAG12-ipt* plants than in NT plants during drought stress [3–4]. A stimulation of glycolysis is thought to be a beneficial early drought stress tolerance mechanism, as also evident in another resurrection plant *Craterostigma plantagineum* [47], for readily available free sugars for osmotic adjustment and energy production. This increased flux through glycolysis may play a role in CK regulation of drought tolerance.

The transcript levels of several transporters were greater in the *SAG12-ipt* line compared to NT plants under drought stress. An aquaporin Pip1-2, water transporting channel, was up-regulated by drought stress in *SAG12-ipt* plants. A greater expression of aquaporin transcripts occurred in drought-tolerant lines compared to a drought sensitive cultivar of chickpea (*Cicer arietinum*) [48]. The up-regulation of aquaporins during drought stress has been reported in Arabidopsis [49] and rice [50]. In addition to water movement, aquaporins also play an important role in mobilizing other metabolites throughout the plant, including those of small molecular weight such as glycerol and urea and gases such as NH_3_ and CO_2_ [51]. Metabolites that were important in the drought response in *SAG12-ipt* plants included several small molecular weight compounds including glycerol. The up-regulation of these transports may play an important role in allowing water and metabolite movement during stress periods. The maintenance of greater RWC in *SAG12-ipt* plants than the NT under drought stress could be related to this type of differential aquaporin or transporter expression.

Another type of transporter, an ABC transporter was found to be up-regulated in NT plants. ABC transporter transcripts were greater in tall fescue plants sensitive to drought stress [14]. Other transcripts that were increased by drought stress in NT plants but not in *SAG12-ipt* plants include one coding for a CDGSH iron sulfur domain 1 protein and a vacuolar ATPase. CDGSH iron sulfur domains are typically located in mitochondrial membranes, serving as transport channels for electron gradient regulation and iron transport [52]. Vacuolar ATPases are primarily involved in transporting ions across the plasma membranes such as Ca^2+^ ions [53]. The requirement of up-regulation of these transcripts could be related to the need for maintenance of osmotic balance or osmotic adjustment in plants exposed to drought stress.

Transcripts coding for proteins related to protein transport or protein degradation were the main types of transcripts differentially regulated in the protein destination/storage category and more changes in these transcripts were detected in NT plants than in *SAG12-ipt* in response to drought. For example, an ATP-dependent Clp protease ATP-binding subunit clpA and several transcripts associated with ubiquitin were found to be either up- or down-regulated in NT plants. The ATP-dependent Clp protease ATP-binding subunit clpA homolog may interact with a clpP-like protease involved in degradation of denatured proteins in the chloroplast [54]. Ubiquitin pathways mark proteins for degradation by the proteasome or can function to regulate membrane bound proteasome associated signaling transduction caused by CK during cell division processes [55]. Stability of ubiquitin transcripts have been associated with drought tolerance in a dessication tolerant grass species (*Sporobolus stapfianus*). Transcripts coding for an ubiquitin-conjugating protein was found to be down-regulated due to drought stress in barley (*Hordeum vulgare*) [30]. Tian et al. [56] found the ubiquitin proteasome pathway to be up-regulated under heat stress conditions in a grass species with superior heat tolerance. Alternate mechanisms could be employed in regard to protein degradation and stress tolerance since increased accumulation of proteases is important for stress tolerance to facilitate utilization of nutrients in protein turnover whereas a reduction of protease activity could allow for maintenance of protein health and functionality under stress. Transcripts such as GTPase SAR1 are necessary for the initiation of the process of transporting proteins from the exit sites of the Golgi apparatus [57]. This could reflect the requirement of newly synthesized proteins to replace those damaged by drought stress.

Leu-rich repeat (LRR) receptor kinases were decreased in response to drought stress in NT plants but exhibited enhanced expression in *SAG12-ipt*. LRR receptor kinases are a large group of transmembrane receptor proteins that have diverse functions in developmental and defense related processes. LRRs are typically localized to plasma membranes and a limited number of them can be up-regulated by increased ABA content [58]. LRR receptor kinases function is also closely tied to regulation by peptide hormones (e.g. phytosulfokine) that are less well understood [59]. Differential expression of LRR in NT and *SAG12-ipt* plants suggested the involvement of this gene in the signal transduction process for the differential drought responses of the two plant types.

A universal stress protein (USP) was detected in NT plants due to drought stress in SSH analysis, but more USP transcripts were detected in *SAG12-ipt* plants. An accumulation of the USP 1 transcripts was also detected due to drought stress in rice [60] and is responsive to other abiotic stress such as cold [35]. Different studies have found contrasting results regarding the expression or accumulation of USP proteins as they relate to drought sensitivity or tolerance [35, 60–61]. Species and stress duration and differences in USP isoforms may be the cause of whether an up-regulation or a down-regulation of gene transcripts and differences in USP protein accumulation are correlated with stress tolerance. Similar to our results, various USP genes were more highly expressed in the salt sensitive variety compared to the tolerant type [61]. USP transcripts and protein generally has been associated with stress incidence and accumulate in the cytoplasm when cells are undergoing stress damage [62–63]. Therefore, the NT plants may be up-regulating the USP protein due to cellular damage. Genetic manipulation of USP proteins may be a viable method to improve plant stress tolerance [62]. How CK may regulate USP proteins is not conclusive from our studies. However, since the response occurred in the more drought-sensitive NT plants, further investigation into the responses of USPs in turfgrass species may be warranted and enhancement of the response of USP under drought stress in grass species may help promote drought tolerance.

Transcripts coding for an isoflavone reductase-like protein 5 were found to be greater in *SAG12-ipt* plants compared NT under drought stress. Isoflavone reductase proteins are known to decrease due to drought stress [64]. Maintenance of greater levels of isoflavone reductase enzymes is associated with drought tolerance [65]. Our results are consistent with those found in loblolly pine (*Pinus taeda*), where isoflavone transcripts were up-regulated in response to mild drought stress but not in the severe stress state [66]. Thus, this suggests less stress damage in *SAG12-ipt* plants at the same level of cellular water deficit than in NT plants, which is consistent with our physiological results [3–4]. Transcripts encoding iosoflavone reductase were also found to exhibit differential regulation in SSH libraries constructed comparing differential tall fescue cultivars in response to heat stress [14]. Transcripts coding for a putative dipthine synthase enzyme were down-regulated by drought stress only in NT plants. However, not much information is available regarding dipthine synthase and drought tolerance in plants.

In conclusion, genes of particular interest in how CK may enhance the drought tolerance response were primarily coding for proteins associated with major metabolic functions such as those regulating energy production, metabolism, and stress defense. Further studies evaluating the downstream effects of differentially expressed genes discussed here that were associated with CK maintenance under drought stress in creeping bentgrass may be beneficial for further understanding of how these genes may relate to CK regulation under drought stress in creeping bentgrass.

## Abbreviations

NT: null transformant plant line
WT: wild-type
*SAG12*: senescence activated promoter 12
*ipt*: isopentenyl transferase gene
RWC: relative water content
GAPDH: glyceraldehyde-3-phosphate dehydrogenase
RuBisCO: ribulose-1,5-bisphosphate carboxylase/oxygenase large subunit
CAT: catalase
OEE: oxygen evolving enhancer
LRR: Leucine-rich-repeat
CK: cytokinin
SSH: suppression subtractive hybridization
